# Recover recovery style from psychosis: a psychometric evaluation of the German version of the Recovery Style Questionnaire (RSQ)

**DOI:** 10.1017/S2045796018000471

**Published:** 2018-09-11

**Authors:** M. Gruber, T. Rumpold, B. Schrank, I. Sibitz, B. Otzelberger, R. Jahn, M. Amering, A. Unger

**Affiliations:** 1 Division of Social Psychiatry, Department of Psychiatry and Psychotherapy, Medical University of Vienna, Austria; 2 Department of Radiation Oncology, Comprehensive Cancer Center Medical University of Vienna, Waehringer Straße 18-20, 1090 Vienna, Austria; 3 Department of Adult Psychiatry, Karl Landsteiner University of Health Sciences, University Clinic Tulln, Austria; 4 Institute of Psychology, University of Vienna, Austria

**Keywords:** Psychosis, recovery style questionnaire, schizophrenia, validation

## Abstract

**Aims:**

The way an individual handles the experience of psychosis, the so-called ‘recovery style’, has been shown to substantially affect long-term outcomes. The Recovery Style Questionnaire (RSQ) measures this psychological dimension. The aim of this study was to provide a validation of the German version of the RSQ and to raise awareness for recovery-oriented approaches.

**Methods:**

The RSQ was translated into German according to the guidelines of the WHO and patients were administered this questionnaire and measures of internalised stigma, psychotic symptoms, illness concept, empowerment, self-esteem and quality of life. Descriptive statistics were demonstrated to characterise the sample. Reliability was assessed in different forms: internal consistency, test–retest reliability and split-half reliability. Items were evaluated with descriptive data and item-total correlations. Convergent and discriminant validity were shown, and a confirmatory factor analysis was performed. In order to ameliorate the model, a *post hoc* model modification was done.

**Results:**

The sample consisted of 138 patients diagnosed with schizophrenia spectrum disorders (mean age: 35.7 years; 53.6% men; mean duration of illness: 20.6 years) with a mean RSQ overall percentage of 66.12 (s.d. ± 17.43%), mainly representing the categories ‘mixed picture’ and ‘tends towards integration’. The reliability of the RSQ was acceptable with a Cronbach's *α* of 0.741 and a test–retest coefficient of 0.502. Item-total correlations were not acceptable for 27 of 39 items. Moderate evidence for convergent validity of the RSQ was found. Confirmatory factor analysis revealed that the 13-factor model with 39 items originally proposed was partially poorly replicated in the present sample (*χ*^2^ ratio to degrees of freedom (*χ*^2^/df) of 1.732, Comparative Fit Index (CFI) of 0.585, Normed Fit Index (NFI) of 0.414, Tucker–Lewis Index (TLI) of 0.508, root mean square error of approximation (RMSEA) of 0.095). The RSQ was modified based on item-total correlations and path coefficients of the single items. The confirmatory factor analysis of the resulting one-factor model with 11 items showed adequate fit to the data (*χ*^2^/df of 1.562, CFI of 0.936, NFI of 0.847, TLI of 0.910, RMSEA of 0.083) and demonstrated good model fit.

**Conclusions:**

Despite partially insufficient psychometric data of the original RSQ, the concept of recovery style is beneficial to psychiatric research and clinical practice. The underlying idea is valuable, and the questionnaire needs further development. Therefore, a short version of the RSQ is proposed.

## Introduction

A lot of recent research efforts have been devoted to prodromal and first-episode symptoms of schizophrenia spectrum disorders; however, the long-term outcome and handling of these complex disorders also deserve attention. Recovery from schizophrenia is possible and approximately 40% of individuals affected have positive outcomes in occupational and social domains (Jääskeläinen *et al*., 2013). Although the associated stigma has not subsided sufficiently from society or the health care system, the concept of recovery from schizophrenia has evolved and is finding growing acceptance (Slade *et al*., 2012; Norman *et al*., 2017). Recovery-oriented approaches have been recognised as best suited for psychiatric rehabilitation in severe mental disorders (Xu *et al*., 2018).

The concept of recovery style provides an important approach to understand how individuals handle psychosis (Espinosa *et al*., 2016). It has been proposed that people recovering from psychosis adopt one of two distinct recovery styles: either they ‘seal-over’ by avoiding the psychotic experience, not seeing it as a part of themselves or they ‘integrate’ by incorporating the psychotic episode into their identity (McGlashan *et al*., 1976; 1977). Recovery style has been demonstrated to influence treatment engagement and illness status (Tait *et al*., 2003).

‘Sealing-over’ recovery style is associated with insecure identity and little resilience (Drayton *et al*., 1998; Tait *et al*., 2004). These individuals more often have negative experiences in early attachment as well as current social difficulties. This might explain why these patients seek less contact with services and have a higher rate of involuntary measures (Tait *et al*., 2004; O'Donoghue *et al*., 2011). A weaker therapeutic alliance was observed to be more frequent in patients with a sealing-over recovery style (Cavelti *et al*., 2016). ‘Sealing-over’ recovery style was also associated with more predominant negative symptoms and more overall illness severity. Higher levels of thought disorders may interfere with the patient's capability to understand experiences and predicted rather avoidant coping strategies (Cavelti *et al*., 2016).

‘Integration’ is the more favourable recovery style. In presence of the same severity of illness, an integrative recovery style may indicate higher service engagement (Tait *et al*., 2003; Vender *et al*., 2014). In patients with severe mental illness in community services and long-term secure services, not recovery style but insight directly influenced the type of treatment service. However, insight was closely related to recovery style (Fitzgerald, 2010). A moderator role of recovery style between internalised stigma and emotional distress was found in people with persecutory delusions. People with ‘integration’ recovery styles, even if feeling stigmatised, tend to be more resilient to depression (Espinosa *et al*., 2016). Nevertheless, in individuals with recent onset of psychosis and post-traumatic stress disorder syndrome, a trend towards an integrative coping style was found (Mueser *et al*., 2010).

To identify these distinct recovery styles, two instruments have been developed for research purposes and clinical use. The ‘Integration Sealing over Scale’ (ISOS, McGlashan, 1987) is an extensive semi-structured clinical interview requiring rating by a clinician. The ‘Recovery Style Questionnaire’ (RSQ; Drayton *et al*., 1998) has been developed as a short self-report version of the ISOS. Initial psychometric evaluation of the RSQ by the developers showed good internal consistency, test–retest reliability, face- and criterion-related validity with the ISOS (Drayton *et al*., 1998). Sound psychometric parameters were also reported by others (Modestin *et al*., 2009; Poloni *et al*., 2010; Nasillo *et al*., 2013).

However, efforts of further scale validation have been scarce (Cavelti *et al*., 2012) and a factorial validity assessment of the RSQ has so far never been conducted to the best of our knowledge (Grinter, 2012). Furthermore, a German instrument to measure recovery style in psychosis is needed. Therefore, the aim of this study was to psychometrically re-evaluate the RSQ during the process of developing a German version of the questionnaire.

## Methods

### Participants

The participants were recruited from psychiatric inpatient and outpatient treatment units. Patients included were (a) aged between 18 and 65, (b) had a diagnosis of a schizophrenia spectrum disorder according to ICD-10, (c) gave written informed consent and (d) able to understand the German language sufficiently. Exclusion criteria were (a) severe cognitive impairment with serious impairment or inability to communicate, (b) seriously impaired judgement and reality testing as in the case of an acute psychotic episode.

### Procedures

Data were collected at the Department of Psychiatry and Psychotherapy at the Medical University of Vienna and a collaborating public mental health centre in Vienna. Recruitment was done by psychiatric residents or psychiatric consultants. Patients received detailed information about the study and were able to ask questions concerning the project. Those willing to participate signed informed consent. The presence of psychotic symptoms and medical data were asked for, and the questionnaires mentioned below were administered.

### Instrument

The RSQ (Drayton *et al*., 1998, see Table 1) consists of 39 items divided into 13 subscales each comprising three separate questions (see Table 2). The patient rates each item with either ‘agree’ or ‘disagree’. Using a coding frame, each answer is identified as either ‘sealing-over’ (1 point) or ‘integration’ (2 points). Each of the 13 subscales is rated separately (3–4 points ‘sealing-over’ and 5–6 points ‘integration’) and finally a formula is used to calculate the overall percentage of integration: (RSQ overall percentage = number of scales with overall rating of integration/13 × 100%). From the RSQ overall percentage, the following categories are built: 0–17% = 6 sealing-over, 18–33% = 5 tends towards sealing-over, 34–49% = 4 mixed picture, sealing-over predominates, 50–67% = 3 mixed picture, integration predominates, 68–82% = 2 tends towards integration, 84–100% = 1 integration. The initial scale development paper (Drayton *et al*., 1998) reported good psychometric properties with Cronbach's *α* of 0.73, test–retest reliability of 0.81 and suggested two recovery styles (i.e. sealing-over and integration) composed of 13 factors, without reporting a factor analysis.

**Table 1.** Items of the Recovery Style Questionnaire with valid answers (*n*) and item-total correlation *r*_it_

Items	*N*	Answer Yes (%)	Answer No (%)	Item-total correlation *r*_it_
1. There was a gradual build-up to me becoming ill.	136	72.8	27.2	**0.242**
2. My illness is not a part of my personality.^a^	134	55.2	44.8	0.045
3. I am responsible for what I think when I am ill.	136	51.5	48.5	**0.262**
4. I am not interested in my illness.^a^	134	77.6	22.4	0.126
5. My illness taught me new things about myself.	136	83.8	16.2	**0.302**
6. I need help to solve the problems caused by my illness.	137	86.9	13.1	0.071
7. My illness was caused by my difficulties in coping with life.	135	71.1	28.9	0.063
8. I have had a nervous breakdown.	132	56.1	43.9	0.056
9. I can see positive aspects to my illness.	138	58.7	41.3	**0.486**
10. My illness had a strong impact on my life.	138	92.0	8.0	0.106
11. I am not frightened of mental illness.	135	34.1	65.9	0.158
12. I liked some of the experiences I had when I was ill.	134	47.0	53.0	**0.430**
13. My illness has helped me find a more satisfactory life.	130	54.6	45.4	**0.434**
14. My illness came on suddenly and went suddenly.^a^	137	76.6	23.4	**0.250**
15. My illness is a part of me.	136	71.3	28.7	−0.002
16. I am not responsible for my actions when I am ill.^a^	129	58.9	41.1	**0.358**
17. I am curious about my illness.	136	87.5	12.5	0.184
18. I understand myself better because of my illness.	133	65.4	34.6	**0.256**
19. I can manage the problems caused by my illness, alone.^a^	131	73.3	26.7	−0.141
20. Others are to blame for my illness.^a^	135	63.0	37.0	−0.090
21. I have had a medical illness.^a^	133	32.3	67.7	−0.054
22. Nothing good came from my illness.^a^	134	50.7	49.3	**0.538**
23. My illness has had little effect on my life.^a^	128	92.0	8.0	**0.248**
24. I am frightened of mental illness.^a^	135	34.1	65.9	**0.203**
25. I didn't like any of the unusual experiences I had when I was ill.^a^	134	47.0	53.0	**0.399**
26. It's hard to find satisfaction with life, since I was ill.^a^	130	54.6	45.4	**0.271**
27. My illness came on very suddenly.^a^	138	60.1	39.9	0.193
28. My illness is alien to me.^a^	134	79.1	20.9	**0.290**
29. I am responsible for my thoughts and feelings when I am ill.	134	59.7	40.3	0.166
30. I don't care about my illness now that I am well.^a^	128	80.5	19.5	**0.363**
31. I want to be the person I was before my illness.^a^	135	44.4	55.6	**0.280**
32. Others can help me solve my problems.	136	91.2	8.8	0.173
33. My illness was caused by stress in my life.	131	73.3	26.7	**0.202**
34. I have suffered an emotional break-down.	131	74.8	25.2	0.085
35. Being ill had good parts too.	136	47.8	52.2	**0.577**
36. I am not really interested in my illness.^a^	133	84.2	15.8	**0.307**
37. I liked some of the unusual ideas I had when I was ill.	131	48.1	51.9	**0.434**
38. My life is more satisfying since my illness.	126	37.3	62.7	**0.382**
39. My attitude toward mental illness is better now, than before I was ill.	131	83.2	16.8	0.081

a Inverted items, bold: item-total correlation ⩾0.20, items selected via model modification after confirmatory factor analysis.

**Table 2.** Definitions of domains of the Recovery Style Questionnaire and Cronbach's *α* values

Domain	Associated items	Definition	Cronbach's *α*	Number
I. *Continuity*	1, 14, 27	If a continuity between psychosis and emotional conflicts after and prior to psychosis is seen by the individual	0.745^b^	136
II. *Ownership*	2, 15, 28	If the psychosis is seen as internal or external	0.256	131
III. *Responsibility*	3, 16, 29	If the individual feels responsible for psychotic thoughts and acts	0.721^b^	126
IV. *Curiosity*	4, 17, 30	Concerning the psychosis	0.579^a^	122
V. *Education*	5, 18, 31	If the psychosis is seen as a possibility to learn more about oneself	0.374	128
VI. *Help-Seeking*	6, 19, 32	Does the individual seek help from others in order to manage psychosis	0.360	129
VII. *Blame*	7, 20, 33	Does the individual blame difficulties in his/her life as a cause for psychosis	0.009	127
VIII. *Cause*	8, 21, 34	Does the individual feel he/she has had a nervous breakdown	0.198	124
IX. *Optimism*	9, 22, 35	Ability to identify positive aspects about having a psychosis	0.712^b^	132
X. *Impact*	10, 23, 36	How the impact on life affects the individual	0.003	133
XI. *Fear*	11, 24, 37	Attitude towards mental illness	0.452	128
XII. *Liking*	12, 25, 38	If some of the experiences during psychosis were liked	0.605*	122
XIII. *Satisfaction*	13, 26, 39	Has the individual experienced life fulfilment through the experience of psychosis	0.440	121

a Poor but acceptable Cronbach's *α* ⩾ 0.5.

b Good Cronbach's *α* ⩾ 0.7.

### Translation and back translation

The RSQ was translated into German according to the guidelines of the WHO (Sartorius and Janca, 1996). One of the authors (A.U.) is a German and English native speaker and works as a psychiatrist. She translated the English original version into German and then an English back-translation was done by a German–English professional translator. The English back-translation was reviewed and checked for consistency by one of the authors of the original English version (M.B.; Drayton *et al*., 1998).

### Validation

As recommended by Guadonoli and Velicer (1988) and Costello and Osborne (2005), about 150 cases were planned to be appropriate when performing a factor analysis on the 39-item RSQ. The correlation coefficient should become an adequate estimator of the population correlation coefficient when sample sizes reach this level. To establish test–retest reliability with a power of 0.80 and an *α* of 0.05, assuming that at least 10% of the sample agrees with the item and considering the possibility that occasionally items might be missing, we found a sample of 30 patients to be suitable to fill out the questionnaire again after a couple of weeks. For the assessment of convergent and discriminant validity, other instruments with related or distinguishing constructs were administered. The Internalised Stigma of Mental Illness inventory (ISMI) (Ritsher *et al*., 2003; German Version: Sibitz *et al*., 2013) was developed to measure internalised stigma. The 27-item scale consists of the subscales alienation, discrimination experience, social withdrawal, stereotype endorsement and resistance. The German version of the ISMI showed good psychometric properties (Sibitz *et al*., 2013). The well-established Positive and Negative Syndrome Scale (PANSS) (Kay *et al*., 1987) was used. The Illness Concept Scale (KK-Scale) (Linden *et al*., 1988) is a German questionnaire, also named ‘Krankheitskonzept Skala’ (KK-Scale). It consists of 29 items and was designed for schizophrenic patients. The scale assesses patients’ illness-related attitudes. Chronbach's *α* and retest–test reliability were moderate (Linden *et al*., 1988). The 28-item Rogers Empowerment Scale (Rogers *et al*., 1997) measures empowerment in five different dimensions: self-esteem and self-determination, power *v.* feeling powerless, autonomy, optimism and control over the future, justified anger. The scale demonstrated excellent reliability and validity in large samples (Rogers *et al*., 2010). The revised version of the self-esteem scale by Rosenberg (1965) is a ten-item self-rating instrument measuring positive and negative feelings about the self. It has been validated in German language and showed good psychometric properties (Von-Collani and Herzberg, 2003). The well-established WHOQOL-BREF is a 26-item short version of the WHOQOL-100 instrument with satisfactory internal consistency (WHO, 1996). It entails the dimensions physical well-being, psychological well-being, social relations and the environment.

### Statistical analysis

The statistical analysis was calculated using the software packages SPSS® 24 and AMOS^®^ 24. Descriptive statistics are presented in absolute numbers and percentages. Internal consistency of the RSQ was calculated and corrected for dichotomous variables by the Kuder–Richardson Formula 20 (Kuder and Richardson, 1937). A Cronbach's *α* coefficient >0.70 was considered acceptable. Pearson correlation coefficient was used to evaluate test–retest reliability. Additionally, split-half reliability was calculated with the Spearman–Brown coefficient. The convergent and discriminant validity were examined with correlational analyses of the RSQ overall percentage score with other constructs.

A confirmatory factor analysis was performed to test the fit of our data to a 13-factor model deriving from the original 13-subscale structure of the English RSQ with 39 dichotomous items (Drayton *et al*., 1998). The acceptability of the model was judged by following recommended standards: *χ*^2^ ratio to degrees of freedom (*χ*^2^/df) < 2.00, Comparative Fit Index (CFI), Normed Fit Index (NFI) and Tucker–Lewis Index (TLI) > 0.90 and the root mean square error of approximation (RMSEA) values of 0.06 or less for a good fit and 0.08 or less for a reasonable fit (Bentler, 1990; Backhaus *et al*., 2006; Schreiber, 2008; Moosbrugger and Kelava, 2012). To compute fit indices in the CFA, estimates of standardised regression weights were used because the weight gives information about the implication that each item should preferably have. They can be interpreted like effect sizes following the effect size classification by Cohen (1988).

## Results

### Sample characteristics

Of 251 persons approached, 94 declined to participate, 16 dropped out because they did not meet the inclusion criteria and three patients did not complete the RSQ. The study sample comprised 138 participants, 56 of them missed at least one RSQ item. Therefore, only 82 cases could be included into some of the calculations (e.g. internal consistency). In Table 3, socio-demographic and illness-related data of the sample are shown. Overall, 74 men (53.6%) and 64 women (46.4%) aged between 19 and 69 years (M 36.4, s.d. ± 11.3) were included. Ninety-one persons (65.9%) were diagnosed with paranoid schizophrenia (WHO, 1993) (ICD-10: F20.0), three (2.2%) with hebephrenic schizophrenia (ICD-10: F20.1), one (0.7%) with undifferentiated schizophrenia (ICD-10: F20.3), three (2.2%) with residual schizophrenia (ICD-10: F20.5), four (2.9%) with schizotypal disorder (ICD-10: F21), three (2.2%) with persistent delusional disorder (ICD-10: F22), two (1.5%) with acute polymorphic psychotic disorders (ICD-10: F23.1) and 31 (22.5%) with schizoaffective disorder (ICD-10: F25). Most participants had been suffering from a psychosis of the schizophrenic spectrum for several years (M 15.1, s.d. ± 15). The patients were moderately to severely ill (PANSS M 72.9, s.d. ± 12.6) and demographic characteristics reflected the typical adverse effects of this illness: 110 participants were unmarried (79.7%) and more than half (*n* = 52, 52.9%) lived alone. Nearly half of all participants (*n* = 65, 47.1%) received invalidity pension and only three people had a paid work.

**Table 3.** Socio-demographic and illness-related data

	*N* = 138
Age, years (average and s.d.)	(36.4 ± 11.3)
Gender (N, %)	
Women	64 (46.4%)
Men	74 (53.6%)
In treatment at	
Day clinic	34 (24.6%)
Outpatient clinic	42 (30.4%)
Inpatient	62 (44.9%)
Marital status (N, %)	
Unmarried	110 (79.7%)
Married or having a live-in partner	15 (10.9%)
Separated or divorced	13 (9.4%)
Have a partner (N, %)	40 (29.0%)
Number of friends (average and s.d.)	4.0 (±8.7)
Social network (N, %)	
No or few social contacts	39 (28.3%)
Few friends, short-term friendships	34 (24.6%)
Enough friends	63 (45.7%)
Unknown	2 (1.4%)
Living situation (N, %)	
Living alone	73 (52.9%)
With parents	31 (22.5%)
With partner	22 (15.9%)
Apartment-sharing community	8 (5.8%)
Assisted living	4 (2.9%)
Highest education level (N, %)	
Basic education	23 (16.7%)
Vocational school, trade school	32 (23.2%)
High school diploma	35 (25.4%)
University	48 (34.8%))
Means of subsistence (N, %)	
Work	3 (2.2%)
Sick leave	30 (21.7%)
Retired	65 (47.1%)
Student	7 (5.1%)
Public social assistance	13 (9.4%)
Unemployment compensation	8 (5.8%)
Other	10 (13.8%)
Unknown	2 (1.4%)
Diagnosis (N, %)	
Paranoid schizophrenia (ICD-10: F20.0)	91 (65.9%)
Hebephrenic schizophrenia (ICD-10: F20.1)	3 (2.2%)
Undifferentiated schizophrenia (ICD-10: F20.3)	1 (0.7%)
Residual schizophrenia (ICD-10: F20.5)	3 (2.2%)
Schizotypal disorder (ICD-10: F21)	4 (2.9%)
Persistent delusional disorder (ICD-10: F22)	3 (2.2%)
Acute polymorphic psychotic disorders (ICD-10: F23.1)	2 (1.5%)
Schizoaffective disorder (ICD-10: F25)	31 (22.5%)
PANSS (average and s.d.)	72.9 (±12.6)
Age at onset of illness (average and s.d.)	20.6 (±10.3)
Duration of illness (average and s.d.)	15.2 (±15.0)
Number of hospital stays (average and s.d.)	5.9 (±6.5)

Standard deviation s.d.

**Fig. 1.**
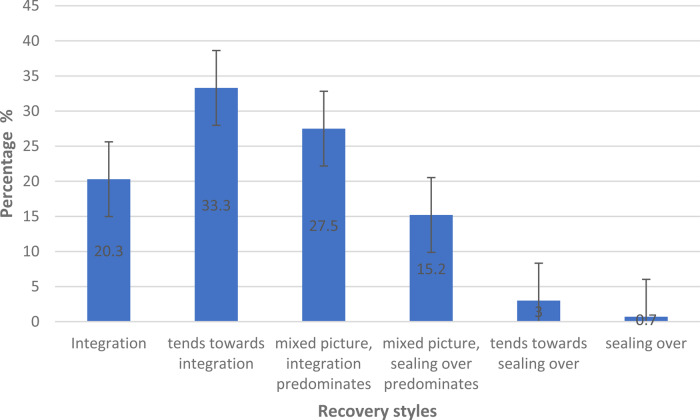
Distribution of recovery styles, percentages with standard errors, *N* = 138.

Out of 138 participants, the mean RSQ overall percentage of integration in our sample was 66.12% (s.d. ± 17.43), representing the category ‘mixed picture, integration predominates’. The majority of participants showed either a ‘mixed picture where integration predominates’ (*n* = 38, 27.0%), ‘tends towards integration’ (*n* = 46, 32.6%) or ‘integration’ (*n* = 28, 19.9%). Few participants showed either ‘mixed picture where sealing-over predominates’ (*n* = 21, 14.9%), ‘tend towards seal-over’ (*n* = 4, 2.8%) or ‘sealing-over’ (*n* = 1, 0.7%) (see Fig. 1).

### Reliability

Eighty-two questionnaires with no missing items were included for internal consistency. An acceptable Cronbach's *α* of 0.741 was achieved. Most of the subscales performed below an acceptable range (see Table 1). The test–retest coefficient for the RSQ overall per cent scores (*n* = 24) was *r* = 0.502 for 52.17 days with a significance of *p* = 0.01 (two-tailed). The split-half reliability for the 39 RSQ items yielded a Spearman–Brown coefficient of 0.686 with *n* = 82.

### Descriptive statistical evaluation of items

The RSQ consists of 39 items, 17 items were inverted during questionnaire development (Drayton *et al*., 1998). Table 1 shows the formulation of each individual item. It was found that the proportion of integrative answers is lower for items with negative formulations. The 17 negatively poled items were answered by 38.0% of the participants with integrative patterns, while the 22 positively poled items of 65.8% were given an integrative answer. Respondents rejected items like item 23, ‘My illness has had little effect on my life’ or item 4 ‘I am not interested in my illness’. In addition, separation powers were calculated for the individual items (see Table 1). The power of separation indicates how well a single item distinguishes persons with a ‘sealing-over’ and an ‘integrative recovery style’. Corrected separations *r*_it_ of ⩽0.20 were considered too low. Only 22 items showed satisfactory values. For example, item 19, ‘I can manage the problems caused by my illness, alone’ was not clearly distinguishable between the two types of recovery styles.

### Convergent and discriminant validity

The convergent and discriminant validity were examined with correlations of the RSQ overall percentage score with other constructs. The RSQ was positively correlated with psychological quality of life (*r* = 0.323; *p* < 0.01) and showed an overlap with following concepts: resistance on the internalised stigma scale (*r* = 0.389; *p* < 0.01), empowerment (*r* = 0.422; *p* < 0.01) and specifically optimism on the empowerment scale (*r* = 0.428; *p* < 0.01). There was a positive relationship between the recovery style and the construct of self-esteem, on the self-esteem scale (*r* = 0.324; *p* < 0.01) and self-esteem on the empowerment scale (0.381; *p* < 0.01). A negative association of the RSQ with alienation on the internalised stigma scale was found (−0.309; *p* < 0.01) (see Table 4).

**Table 4.** Correlation of the RSQ overall percentage with different scales, (*n* = 92, listwise)

Scale/sub-scale	Correlation
Quality of life, global	0.281^a^
Quality of life, psychological	0.323^a^
Internalised stigma resistance	0.389^a^
Internalised stigma alienation	−0.309^a^
Self-esteem scale	0.324^a^
Empowerment – overall	0.422^a^
Empowerment – self-esteem	0.381^a^
Empowerment – optimism	0.428^a^
Empowerment – autonomy	0.164
Concept of illness – overall	0.227^b^
PANSS overall	−0.023
PANSS negative	−0.054
PANSS positive	−0.002
PANSS general	0.001

a Values under 0.01 were considered highly significant.

b Values under 0.05 were considered significant.

### Confirmatory factor analysis

The results of the confirmatory factor analysis are displayed in Supplementary Table 1. The *χ*^2^ test was significant (*p* < 0.001, *χ*^2^ = 1080.604, 624 df) and the *χ*^2^/df of 1.732 demonstrated good model fit, but other indices showed poor model fit with CFI of 0.586, NFI of 0.414 and a TLI of 0.508. The RMSEA of 0.095 indicated reasonable model fit.

### Model modification

**Fig. 2.**
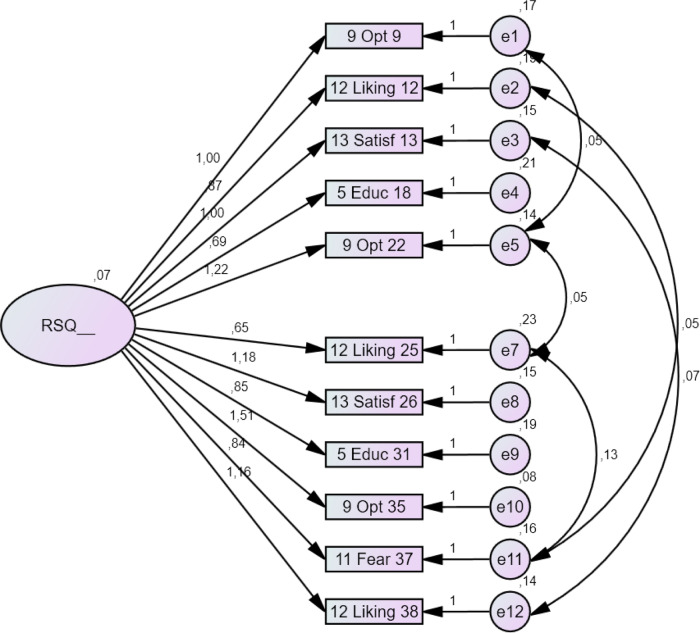
Path diagram of the confirmatory factor analysis of an alternative one-factor model (11 items) with observed and latent variables, left number of rectangle refers to the number of subscale and right number indicates item number; *n* = 82.

The fit to the proposed model was partially poor. In order to ameliorate the model, a *post hoc* model modification was done. It is good practice to assess the fit of each construct and its items individually to determine particularly weak items. Items with low corrected item total correlations (*r*_it_ ⩽ 0.20, see Table 1 and Supplementary Table 2) indicate very high levels of error. These items were removed from the model step by step (Hooper *et al*., 2008). Path coefficients for each item derived from the CFA were also comparable with the results of the item-total correlations (see Supplementary Table S1 and Supplementary Fig. S1). Model modification revealed a one-factor model consisting of 11 items with acceptable fit to the data (see Fig. 2; Supplementary Table S2). *χ*^2^/df of 1.562 and CFI of 0.936, NFI of 0.847 and a TLI of 0.910 demonstrated good model fit. The RMSEA of 0.083 indicated reasonable but better model fit. The remaining items with the best parameters were marked in bold in Table 1.

## Discussion

The aim of the present study was the validation of the RSQ. Overall, the reliability of the RSQ scale was acceptable. Confirmatory factor analysis revealed that the 13-factor model originally proposed was not replicated in the present sample. The RSQ was modified based on item-total correlations and path coefficients, the confirmatory factor analysis of the resulting model showed adequate fit to the data.

Internal consistency of the RSQ showed an acceptable value at Cronbach's *α* = 0.741, similar to the original paper where internal consistency was *α* = 0.73 and other validation papers reporting values from *α* = 0.73 (Poloni *et al*., 2010) to *α* = 0.78 (Modestin *et al*., 2009; Grinter, 2012). However, the internal consistency of most of the subscales was below an acceptable value, which could be expected because each subscale consists only of three items. The test–retest reliability was moderate with a correlation of *r* = 0.502 which translates to only about half of all responses being identical when asked again a second time after a couple of weeks. A previous study has also reported a test–retest reliability correlation coefficient of *r* = 0.50 for the RSQ (Poloni *et al*., 2010), whereas the test–retest reliability for 1 month of the original validation was *r* = 0.81 (Drayton *et al*., 1998).

The sample size of 56 participants in the original study was small with a low proportion of female participants (26.8%) (Drayton *et al*., 1998). In comparison, our study included 138 participants with a balanced gender distribution. In the original paper, only people diagnosed with schizophrenia according to ICD-10 criteria were included. The present sample consisted of a broader diagnostic spectrum with 65.9% diagnosed with paranoid schizophrenia, 2.2% with hebephrenic schizophrenia, 0.7% with undifferentiated schizophrenia, 2.2% with residual schizophrenia, 2.9% with schizotypal disorder, 2.2% with persistent delusional disorder, 1.5% with acute polymorphic psychotic disorders and 22.5% with schizoaffective disorder. The age at onset of illness and the duration of illness were quite similar in both studies but the number of admissions was higher in our investigation with 5.9 hospital stays on average compared with three admissions in the original paper. The present sample was moderately to markedly ill with a mean PANSS score of 72.9 (s.d. 12.6) (Leucht *et al*., 2014). Therefore, the larger sample with a broader range but more severe disorders could have influenced the results.

In summary, the confirmatory factor analysis performed on our data yielded a poor-to-moderate model fit to the original 13-subscale structure with 39 items of the RSQ (Drayton *et al*., 1998). To our knowledge, this is the first study to examine the factor structure of the RSQ by means of factor analysis. In the original paper by Drayton (1998), no factor analysis was conducted. The basis for the formation of 13 subscales was the ISOS (McGlashan *et al*., 1976), an observer report measure requiring the interviewer to assess the patient over 13 concepts pertaining to illness-related attitudes. The RSQ was designed as a short self-report assessment with three questions assigned to each concept presented in the ISOS and therefore a face validity with the ISOS was stated. The criterion-related validity with the ISOS was good achieving similar results for the RSQ (*r* = 0.92; Drayton *et al*., 1998). However, most subscales did perform below an acceptable reliability. The evaluation of the questionnaire is not based on a sum score of the 39 items. Each subscale consisting of three items is rated separately and an overall percentage is calculated which refers to an either integrative or sealing-over recovery style. The 13-scubscale structure did disappoint and this might distort the results. Furthermore, 17 out of 39 items showed disappointing separation power. It seemed that more than a third of negatively worded questions (17 items) were more likely to be answered in a non-integrative pattern. Reversing a proportion of items is often used to reduce the effects of response styles by changing the direction of the item. Although this is an established practice, there is no consensus about its effectiveness (van Sonderen *et al*., 2013). Ebesutani *et al*. (2012) found that non-reverse-worded items were associated with superior psychometric properties relative to the reverse-worded items. Often it makes questions more complex and more difficult to understand. Questions like RSQ item 2 ‘My illness is not a part of my personality’ or item 4 ‘I am not interested in my illness’ may not be well read and understood by respondents. Especially patients suffering from schizophrenia for years often have impaired cognitive functioning with thought disorders. They may lack sufficient attention to carefully read and understand the questions, this might influence the truth of the given answers. In contrast, the dichotomous answer format of the RSQ may be beneficial for this patient group due to a dichotomous thinking style often found in patients with psychotic disorders (Peters *et al*., 2014).

The concept of recovery style was related to the construct of resistance to internalised stigma measured with the ISMI (Ritsher *et al*., 2003). This is consistent with recent findings where recovery style was shown to have a moderator role between internalised stigma and depression in people with persecutory delusions (Espinosa *et al*., 2016). Recovery style was also found to overlap with the concept of empowerment and optimism. Recovery style provides an important approach to understand how individuals handle psychosis in a psychological dimension. The RSQ is continued to be used in recovery-oriented research (Modestin *et al*., 2009; O'Donoghue *et al*., 2011; Vender *et al*., 2014; Espinosa *et al*., 2016) and clinical practice. The ISOS, which is essentially the same construct, is still applied, too (Modestin *et al*., 2009; Cavelti *et al*., 2016). Recovery style has been shown to affect symptoms, treatment and outcome in schizophrenia (McGlashan *et al*., 1987; Modestin *et al*., 2004; 2009; Cavelti *et al*., 2016).

### Strengths and limitations

The study provides a comprehensive and independent psychometric evaluation of the RSQ. To the authors’ knowledge, it is the first study to investigate the factor structure of the RSQ and to compare the underlying construct with an independent concept of stigma internalisation (ISMI). Although the sample size of the present study is relatively small, it is acceptable for factor analytical procedures and it is so far the largest sample to investigate psychometric properties of the RSQ. The composition of the present sample could be a limitation. Patients were in part recruited at an outpatient clinic where mainly patients with a chronic progression of schizophrenia are often repeatedly treated on a rather long-term basis over years. These patients are usually very advanced in their expertise about psychotic experiences and are, in the majority of cases, stabilised due to the extensive treatment and support. This limits the examined population to those who were in treatment at the time of assessment, and it is not known how results might have differed in a ‘real-world’ population of those who are partly out of treatment or completely on their own without support. The influence of duration of illness on recovery style is unclear and further studies are warranted to determine how transition from sealing-over to integration can best be promoted.

## Conclusions

The use of the RSQ in clinical practice might be inspiring and helpful, especially for treatment planning. The RSQ does cover several topics that are clinically relevant, with items such as ‘I have a medical illness’ and ‘My illness had a strong impact on my life’ insight and the impact the illness has on patients’ lives is reflected. Yet, addressing and discussing topics such as showing interest in one's illness, believing in one's influence on the course of the illness and accepting help and support within the therapeutic relationship might facilitate the acceptance of the illness and further the recovery process. The use of the RSQ in mental health practice might provide the therapist and the patient with an indication of areas in need of enhanced therapeutic discussion and support. First, the RSQ can be a helpful tool for measuring the integration of the recovery concept on the consumer side. Second, identifying patients with sealing-over recovery styles might enable professionals to provide more intensive support to those at risk for poorer service engagement. Anyway, considering the results of the present study pointing to statistical and conceptual shortcomings, the RSQ cannot be recommended for use in recovery-oriented research in its original version (Drayton *et al.*, 1998). Therefore, a short version with better psychometric properties is proposed for research use but further scale validation is needed.

## Supplementary material

For supplementary material accompanying this paper visit http://dx.doi.org/10.1017/S2045796018000471. click here to view supplementary material

## Availability of data and materials

The data that support the findings of this study are available for qualified researchers from, but restrictions for academic and not-commercial use apply to the availability of these data. Data are however available from annemarie.unger@meduniwien.ac.at upon reasonable request.

## References

[ref1] Backhaus K, Erichson B, Plinke W and Weiber R (2006) [Multivariate Analysis Methods. An Application-Oriented Introduction], Berlin: Springer.

[ref2] Bentler PM (1990) Comparative fit indexes in structural models. Psychological bulletin 107, 238–246. 2320703 10.1037/0033-2909.107.2.238

[ref3] Cavelti M, Kvrgic S, Beck EM, Kossowsky J and Vauth R (2012) Assessing recovery from schizophrenia as an individual process. A review of self-report instruments. European Psychiatry 27, 19–32. 22130177 10.1016/j.eurpsy.2011.01.007

[ref4] Cavelti M, Homan P and Vauth R (2016) The impact of thought disorders on therapeutic alliance and personal recovery in schizophrenia and schizoaffective disorder: an exploratory study. Psychiatry Research 239, 92–98. 27137967 10.1016/j.psychres.2016.02.070

[ref5] Cohen J (1988) Statistical Power Analysis for the Behavioral Sciences. 2nd Edn. Hillsdale, NJ: Erlbaum.

[ref6] Costello AB and Osborne JW (2005) Best practices in exploratory factor analysis: four recommendations for getting the most from your analysis. Practical Assessment, Research & Evaluation 10, 1–9.

[ref7] Drayton M, Birchwood M and Trower P (1998) Early attachment experience and recovery from psychosis. British Journal of Clinical Psychology 37, 269–284. 10.1111/j.2044-8260.1998.tb01385.x 9784883

[ref8] Ebesutani C, Drescher CF, Reise SP, Heiden L, Hight TL, Damon JD and Young J (2012) The loneliness questionnaire-short version: an evaluation of reverse-worded and non-reverse-worded items via item response theory. Journal of Personality Assessment 94, 427–437. 22404209 10.1080/00223891.2012.662188

[ref9] Espinosa R, Valiente C, Rigabert A and Song H (2016) Recovery style and stigma in psychosis the healing power of integration. Cognitive Neuropsychiatry 21, 146–155. 26924174 10.1080/13546805.2016.1147427

[ref10] Fitzgerald MM (2010) Comparison of recovery style and insight of patients with severe mental illness in secure services with those in community services. Journal of Psychiatric and Mental Health Nursing 17, 229–235. 20465772 10.1111/j.1365-2850.2009.01498.x

[ref11] Grinter DJ (2012) *Non-engagement in psychosis: a narrative analysis of service-users’ experiences of relationships with mental health services*. (Clinical Psychology thesis). University of Glasgow.

[ref12] Guadagnoli E and Velicer WF (1988) Relation of sample size to the stability of component patterns. Psychological bulletin 103, 265–275. 3363047 10.1037/0033-2909.103.2.265

[ref13] Hooper D, Coughlan J and Mullen MR (2008) Structural equation modelling: guidelines for determining model Fit. Electronic Journal of Business Research Methods 6, 53–60.

[ref14] Jääskeläinen E, Juola P, Hirvonen N, McGrath JJ, Saha S, Isohanni M, Veijola J and Miettunen J (2013) A systematic review and meta-analysis of recovery in schizophrenia. Schizophrenia Bulletin 39, 1296–1306. 23172003 10.1093/schbul/sbs130 PMC3796077

[ref15] Kay SR, Fiszbein A and Opler LA (1987) The positive and negative syndrome scale (PANSS) for schizophrenia. Schizophrenia Bulletin 13, 261–276. 3616518 10.1093/schbul/13.2.261

[ref16] Kuder GF and Richardson MW (1937) The theory of the estimation of test reliability. Psychometrika 2, 151–160. 10.1007/BF02289147 18145837

[ref17] Leucht S (2014) Measurements of response, remission, and recovery in schizophrenia and examples for their clinical application. The Journal of Clinical Psychiatry 75, 8–14. 10.4088/JCP.13049su1c.02 24581453

[ref18] Linden M, Nather J and Wilms HU (1988) [Definition, significance and measurement of disease concepts of patients. The disease concept scale for schizophrenic patients]. [Article in German] Fortschritte der Neurologie und Psychiatrie 56, 35–43. 10.1055/s-2007-1001215 2896142

[ref19] McGlashan TH, Docherty JP and Siris S (1976) Integrative and sealing-over recoveries form schizophrenia: distinguishing case studies. Psychiatry 39, 325–338. 996183 10.1080/00332747.1976.11023903

[ref20] McGlashan TH, Wadeson HS, Carpenter WT Jr and Levy ST (1977) Art and recovery style from psychosis. Journal of Nervous and Mental Disorders 164, 182–190. 10.1097/00005053-197703000-00004 320289

[ref21] McGlashan TH (1987) Recovery style from mental illness and long-term outcome. Journal of Nervous and Mental Disorders 175, 681–685. 10.1097/00005053-198711000-00006 3681279

[ref22] Modestin J, Soult J and Malti T (2004) Correlates of coping styles in psychotic illness. Psychopathology 37, 175–180. 15237247 10.1159/000079421

[ref23] Modestin J, Caveng I, Wehrli MV and Malti T (2009) Correlates of coping styles in psychotic illness – an extension study. Psychiatry Research 168, 50–56. 19457559 10.1016/j.psychres.2008.03.008

[ref24] Moosbrugger H and Kelava A (2012) Testtheorie und Fragebogenkonstruktion. Berlin, Heidelberg: Springer-Verlag.

[ref25] Mueser KT, Lu W, Rosenberg SD and Wolfe R (2010) The trauma of psychosis: posttraumatic stress disorder and recent onset of psychosis. Schizophrenia Research 116, 217–227. 19939633 10.1016/j.schres.2009.10.025

[ref26] Nasillo V, Santos JM, Arrufat F and Obiol J (2013) Translation and adaptation of the Recovery Style Questionnaire into Spanish. Interpsiquis. *14th Virtual Psiquiatria.com Congress [unpublished] from: Lemos-Giráldez S, Garcia-Alvarez L, Paino M, … Andresen R (2015)* . Measuring stages of recovery from psychosis. *Comprehensive Psychiatry* 56, pp. 51–58. 10.1016/j.comppsych.2014.09.021 25444077

[ref27] Norman RMG, Li Y, Sorrentino R, Hampson E and Ye Y (2017) The differential effects of a focus on symptoms versus recovery in reducing stigma of schizophrenia. Social Psychiatry and Psychiatric Epidemiology 52, 1385–1394. 28821903 10.1007/s00127-017-1429-2

[ref28] O'Donoghue B, Lyne J, Hill M, O'Rourke L, Daly S and Larkin C…O'Callaghan E (2011) Perceptions of involuntary admission and risk of subsequent readmission at one-year follow-up: the influence of insight and recovery style. Journal of Mental Health 20, 249–259. 21574790 10.3109/09638237.2011.562263

[ref29] Peters E, Moritz S, Schwannauer M, Wiseman Z, Greenwood KE, Scott J, Beck AT, Donaldson C, Hagen R, Ross K, Veckenstedt R, Ison R, Williams S, Kuipers E and Garety PA (2014) Cognitive biases questionnaire for psychosis. Schizophrenia Bulletin 40, 300–313. 23413104 10.1093/schbul/sbs199 PMC3932080

[ref30] Poloni N, Callegari C, Buzzi A, Aletti F, Beranzini F, Vecchi F and Vender S (2010) [The Italian version of ISOS and RSQ, two suitable scales for investigating recovery style from psychosis] [Article in Italian]. Epidemiologia e Psichiatrica Sociale 19, 352–359. 21322966

[ref31] Ritsher JB, Otilingam PG and Grajales M (2003) Internalized stigma of mental illness: psychometric properties of a new measure. Psychiatry Research 121, 31–49. 14572622 10.1016/j.psychres.2003.08.008

[ref32] Rogers SE, Chamberlin J, Ellison ML and Crean T (1997) A consumer constructed scale to measure empowerment among users of mental health services. Psychiatric Services 48, 1042–1047. 9255837 10.1176/ps.48.8.1042

[ref33] Rogers SE, Ralph RO and Salzer MS (2010) Validation the empowerment scale with a multisite sample of consumers of mental health services. Psychiatric Services 61, 933–936. 20810594 10.1176/ps.2010.61.9.933

[ref34] Rosenberg M (1965) Conceiving the Self. New York: Basic books.

[ref35] Sartorius N and Janca A (1996) Psychiatric assessment instruments developed by the World Health Organization. Social Psychiatry and Psychiatric Epidemiology 31, 55–69. 8881086 10.1007/BF00801901

[ref36] Schreiber JB (2008) Core reporting practices in structural equation modeling. Research in Social and Administrative Pharmacy 4, 83–97. 18555963 10.1016/j.sapharm.2007.04.003

[ref37] Sibitz I, Friedrich ME, Unger A, Bachmann A, Benesch T and Amering M (2013) [Internalized stigma of Schizophrenia: validation of the German version of the Internalized Stigma of Mental Illness-Scale (ISMI)] [Article in German]. Psychiatrische Praxis 40, 82–91. 10.1055/s-0032-1332878 23354628

[ref38] Slade M, Leamy M, Bacon F, Janosik M, Le Boutillier C, Williams J and Bird V (2012) Epidemiology and Psychiatric Sciences 21, 353–364. 22794507 10.1017/S2045796012000133 PMC6998141

[ref39] SPSS Inc. Released (2016) SPSS for Windows, Version 24.0. Chicago, SPSS Inc.

[ref40] Tait L, Birchwood M and Trower P (2003) Predicting engagement with services for psychosis: insight, symptoms and recovery style. British Journal of Psychiatry 182, 123–128. 10.1192/bjp.182.2.123 12562739

[ref41] Tait L, Birchwood M and Trower P (2004) Adapting to the challenge of psychosis: personal resilience and the use of sealing-over (avoidant) coping strategies. British Journal of Psychiatry 185, 410–415. 10.1192/bjp.185.5.410 15516550

[ref42] Van Sonderen E, Sanderman R and Coyne JC (2013) Ineffectiveness of reverse wording of questionnaire items: let's learn from cows in the rain. PLoS ONE 8, e68967. doi: 10.1371/journal.pone.0068967. 23935915 PMC3729568

[ref43] Vender S, Poloni N, Aletti F, Bonalumi C and Callegari C (2014) Service engagement: psychopathology, recovery style and treatments. Psychiatry Journal [published online], doi: 10.1155/2014/249852. PMC3950906 24701559

[ref44] Von-Collani G and Herzberg PY (2003) Eine revidierte fassung der deutschsprachigen Skala zum Selbstwertgefühl von Rosenberg. Zeitschrift für Differentielle und Diagnostische Psychologie 24, 3–7.

[ref45] World Health Organisation (1993) International classification of diseases 10th revision (ICD-10). 2.Auflage. Bern: Huber.

[ref46] World Health Organization (1996) WHOQOL-BREF Introduction, Administration, Scoring and Generic Version of the Assessment: Field Trial Version. [Last accessed 23.04.2018]. Available at http://www.who.int/iris/handle/10665/63529.

[ref47] Xu Z, Lay B, Oexle N, Drack T, Bleiker M, Lengler S, Blank C, Müller M, Mayer B, Rössler W and Rüsch N (2018) Involuntary psychiatric hospitalisation, stigma stress and recovery: a 2-year stud. Epidemiology and Psychiatric Sciences 31, 1–8. 10.1017/S2045796018000021 PMC6998976 29382403

