# Supplementary material for: Recover recovery style from psychosis: a psychometric evaluation of the German version of the Recovery Style Questionnaire (RSQ)
Source: Epidemiol Psychiatr Sci. 2018 Sep 11;29:e4. doi: 10.1017/S2045796018000471 (PMC8061273; doi:10.1017/S2045796018000471)
Supplement: Supplementary file 1 [file epssup.zip › S2045796018000471sup001.docx]

Table S1 Path coefficients and corrected item total correlations (r_it_) (n =82)

| RSQ item Number | Path coefficient | r_it_ |  |
| --- | --- | --- | --- |
| 9 | 0.544 | 0.503 |  |
| 12 | 0.473 | 0.532 |  |
| 13 | 0.578 | 0.515 |  |
| 18 | 0.382 | 0.276 |  |
| 22 | 0.656 | 0.644 |  |
| 25 | 0.347 | 0.455 |  |
| 26 | 0.641 | 0.537 |  |
| 31 | 0.464 | 0.411 |  |
| 35 | 0.830 | 0.718 |  |
| 37 | 0.492 | 0.531 |  |
| 38 | 0.644 | 0.591 |  |
|  |  | 0.84 |  |

Table S2 Factor structure of the original 13-factor model with 39 items of the Recovery Style Questionnaire and after model modification the 1-factor model with 11 items

|  | **13 Factor model (39 items)** | **1 Factor model (11 items)** |
| --- | --- | --- |
| χ2/df | 1.732 | 1.562 |
| P | <0.001 | 0.014 |
| NFI | 0.414 | 0.847 |
| CFI | 0.586 | 0.936 |
| TLI | 0.508 | 0.910 |
| RMSEA | 0.095 | 0.083 |

Figure S1 Bivariate scatterplot of association between path coefficients (x-axis) and corrected item total correlation (y-axis)
